# Is FGF-23 an early indicator of atherosclerosis and cardiac dysfunction in patients with gestational diabetes?

**DOI:** 10.20945/2359-3997000000070

**Published:** 2018-10-01

**Authors:** Dilek Tuzun, Ayten Oguz, Muhammet Naci Aydin, Ergul Belge Kurutas, Onder Ercan, Murat Sahin, Velid Ünsal, Imran Ceren, Ahmet Akçay, Kamile Gul

**Affiliations:** 1 Kahramanmaras Sütçü Imam University Kahramanmaras Sutcu Imam University Faculty of Medicine Department of Endocrinology and Metabolism Kahramanmaras Turkey Kahramanmaras Sutcu Imam University, Faculty of Medicine, Department of Endocrinology and Metabolism, Kahramanmaras, Turkey; 2 Kahramanmaras Sütçü Imam University Kahramanmaras Sutcu Imam University Faculty of Medicine Department of Cardiology Kahramanmaras Turkey Kahramanmaras Sutcu Imam University, Faculty of Medicine, Department of Cardiology, Kahramanmaras, Turkey; 3 Kahramanmaras Sütçü Imam University Kahramanmaras Sutcu Imam University Faculty of Medicine Department of Biochemistry Kahramanmaras Turkey Kahramanmaras Sutcu Imam University, Faculty of Medicine, Department of Biochemistry, Kahramanmaras, Turkey; 4 Kahramanmaras Sütçü Imam University Kahramanmaras Sutcu Imam University Faculty of Medicine Department of Obstetric and Gynecology Kahramanmaras Turkey Kahramanmaras Sutcu Imam University, Faculty of Medicine, Department of Obstetric and Gynecology, Kahramanmaras, Turkey

**Keywords:** FGF-23, myocardial performance index, gestational diabetes mellitus

## Abstract

**Objective:**

Fibroblast growth factor 23 (FGF-23) is a phosphorus-regulating hormone and plays a role in the pathogenesis of myocardial hypertrophy. The aim of this study was to evaluate the association of FGF-23 levels with echocardiographic parameters and insulin resistance (IR) in patients with gestational diabetes.

**Subjects and methods:**

Fifty-four pregnant patients with gestational diabetes mellitus (GDM) (age, 31.12 ± 5.72 years) and 33 healthy pregnant women (age, 29.51 ± 4.92 years) were involved in the study. Fasting insulin, fasting plasma glucose (FPG), lipid profile, oral glucose tolerance test (OGTT), FGF23, echocardiographic parameters, and carotid artery intima-media thickness (CIMT) were evaluated in the two groups.

**Results:**

The two groups were not significantly different in age, sex, body mass index, lipid profile, or blood pressure. Insulin, homeostatic model assessment-insulin resistance (HOMA-IR), FGF-23 levels, CIMT, left ventricular (LV) mass, LV mass index and myocardial performance index (MPI) were significantly higher in the GDM group. HOMA-IR was positively correlated with FGF-23, and insulin was positively correlated with FGF-23. Additionally, FGF-23 was positively correlated with CIMT, LV mass index, and MPI.

**Conclusion:**

Our findings suggest that monitoring serum FGF-23 may be useful as a non-invasive indicator of subclinical atherosclerosis in patients with GDM.

## INTRODUCTION

Gestational diabetes mellitus (GDM) is defined as a glucose intolerance with onset or first recognition occurring during pregnancy (1). It leads to maternal hyperglycemia, endothelial dysfunction, and abnormal regulation of vascular tone (2). GDM has serious adverse perinatal outcomes and increases long-term risk for the development of type 2 diabetes, obesity, and cardiovascular disease in mother and fetus (3).

Tissue Doppler imaging (TDI) was reported as appropriate for evaluating early changes in systolic and diastolic left ventricle (LV) function (4). The myocardial performance index (MPI) is a sensitive parameter widely used to quantitatively assess global ventricular function in a noninvasive way and combines systolic and diastolic intervals. It is related to morbidity and mortality in many cardiovascular diseases (5). MPI has been studied in several cardiac disorders including heart failure, hypertension, and diabetes, and it has been found to predict worsened morbidity and mortality (6-8). Left ventricular hypertrophy (LVH) is a marker of subclinical cardiovascular disease (CVD) and is associated with cardiovascular morbidity and mortality independent of established risk factors such as age, sex, and diabetes (9). The association between the left ventricular mass index (LVMI) and coronary flow reserve in patients with DM has been investigated in previous studies (10).

Fibroblast growth factor-23 (FGF-23) is a hormone involved in calcium-phosphate homoeostasis, primarily produced and secreted by osteocytes (11). In the renal tubules, FGF-23 binds to the FGF receptor 1c and the klotho coreceptor to promote phosphaturia and lower circulating levels of 1,25-dihydroxyvitamin D (9). FGF-23 may also play a role in some metabolic processes, such as insulin resistance (IR), and may also be a marker of diabetes progression or may increase with diabetes-related complications (12). Moreover, serum FGF-23 was shown to be independently associated with LVH in chronic kidney disease (CKD) (13). It has been investigated in several diseases; however, to the best of our knowledge, the relationship between FGF-23 and myocardial function has not been studied yet in patients with GDM.

Carotid artery intima-media thickness (CIMT) assessment and carotid artery plaque identification using ultrasound are well-recognized tools for identification and monitoring of atherosclerosis. Elevated serum FGF-23 levels have been found to be associated with vascular diseases such as CIMT, arterial stiffness, and coronary atherosclerosis in patients with advanced CKD (14,15).

Therefore, we conducted a cross-sectional study to test the hypothesis that elevated FGF-23 concentrations are independently associated with LVMI, MPI, and CIMT in patients with gestational diabetes and to investigate the correlation between FGF-23 levels and IR in gestational diabetic patients compared to pregnant women with normal glucose tolerance.

## SUBJECTS AND METHODS

This prospective study was conducted between 2014 May and 2015 May in the Department of Endocrinology and Metabolism of Kahramanmaras Sutcu Imam University, Kahramanmaras, Turkey. It was approved by the local ethical committee (Date: 01.08.2013, Decision Number: 2013712-10), and written informed consent was obtained from all subjects.

Sixty-four pregnant patients seen and diagnosed with GDM (the patient group) in endocrinology and metabolism outpatient clinics were included in the study. From the patient group, 10 patients were ruled out due to follow-up failure, and the study continued with 54 patients. Thirty-three consecutive healthy pregnant patients seen in an antenatal obstetrics and gynecology outpatient clinic were chosen as the control group.

### Exclusion criteria

Patients were excluded if they had moderate to severe valvular heart disease, any rhythm other than the normal sinus rhythm, more than a mild degree of pericardial effusion, abnormal left ventricular systolic function (ejection fraction ≤ 50%), known coronary artery disease, clinical suspicion of coronary artery disease, uncontrolled hypertension, or acute illnesses. The patients who had previous impaired fasting glucose, impaired glucose tolerance or diabetes mellitus, family history of diabetes mellitus, chronic and acute renal failure, or a smoking habit and those who used calcium supplements or a vitamin D treatment were also ruled out from the study.

### Study protocol

Fasting plasma glucose (FPG), LDL-cholesterol, HDL- cholesterol, triglycerides (TG), alanine aminotransferase (ALT), calcium, phosphate, insulin, 25 hydroxy vitamin D, parathyroid hormone (PTH), and fibroblast growth factor 23 (FGF-23) were measured at the 24th-28th gestational weeks in all subjects. Blood pressure and anthropometric measurements were performed, and body mass index was calculated. Additionally, an oral glucose tolerance test (OGTT) with 75 g glucose was performed at the 24th-28th gestational weeks in all subjects. Serum glucose levels were evaluated according to American Diabetes Association 2013 criteria (16).

### Laboratory parameters

Blood samples for hormones and biochemical parameters were taken after an overnight fast from an antecubital vein between 08:00 a.m. and 09:00 a.m. Glucose was analyzed by the glucose hexokinase method. LDL, HDL, and TG were analyzed by the turbidymetric method (Siemens Dimension, Clinical Chemistry System, Newark, DE, USA), using appropriate commercial kits. Insulin was determined by a two-site chemiluminescent immunometric assay with solid-phase mouse monoclonal anti-insulin antibodies conjugated with calf intestine alkaline phosphatase in buffer solution (Immulite Insulin, Diagnostic Products Corporations, Los Angeles, CA) with 2 IU/ mL sensitivity. The estimate of insulin resistance by the homeostasis model assessment (HOMA) score was calculated with the formula fasting serum insulin (lU/mL) x fasting plasma glucose (mmol/L)/ 22.5, as described by Matthews and coworkers (17). A HOMA index ≥ 2.5 indicated IR.

### FGF-23 level measurement

Serum FGF-23 levels were determined, and venous blood samples were collected in tubes from the antecubital vein, followed by an overnight fast. The tubes were centrifuged at 2000g (10 min) to remove the serum. Aliquots of serum samples were stored at −80°C until FGF-23 assaying. Serum FGF-23 levels were determined using Human FGF-23 ELISA Kit (cat. number EZHFGF-23-32K) purchased from Millipore (USA) according to the manufacturer's instructions. The Millipore Human FGF-23 ELISA Kit employs the quantitative sandwich enzyme immunoassay technique.

### Echocardiographic assessment

The echocardiographic examinations, including M-Mode, two-dimensional, SDE, and tissue Doppler echocardiography (TDI) recordings, were performed with Vivid 7 Pro (GE, Horten, Norway, 2-4 MHz phased array transducer). Measurements were made according to the American Society of Echocardiography guidelines by a single cardiologist unaware of the clinical data and averaged from five cardiac cycles.

For all participants, two-dimensional, M-Mode, and TDI echocardiography examinations were performed by the same physician. LV mass was calculated with the Devereux Formula (18) and indexed to body surface area.

In all TDI images, the systolic velocity duration was measured as ejection time (ET, ms), the time between the end of the systolic velocity and the beginning of early diastolic velocity was measured as isovolumetric relaxation time (IRT, ms), and the time between the end of the late diastolic velocity and the beginning of systolic velocity as isovolumetric contraction time (ICT, ms) MPI, which is calculated from systolic and diastolic time intervals, reflects the global left ventricular function with good reproducibility, and is independent from the left ventricle's geometry and heart rate.

The myocardial performance index was calculated using the formula (ICT + IRT)/ET.

### Measurement of carotid intima-media thickness

Measurements of all participants' CIMT were performed by the same endocrinologist with the same ultrasound device (Logic P5 System, General Electric Medical Systems, Milwaukee, WI, USA) using a linear transducer of 12 MHz width. The carotid arteries were scanned in longitudinal projection. Intima media is defined as the distance between the beginning of the tunica intima and the beginning of the tunica adventitia. Intima media thickness measurements on the common carotid artery's far wall were bilaterally performed. For reproducible measurements, a high-quality image acquisition was used along a minimum length of 10 mm of an arterial segment. The mean CIMT was calculated from three consecutive examinations.

### Statistical analysis

Statistical analysis was conducted with Statistical Package for Social Sciences 15.0 packet program (SPSS Inc., Chicago, IL). Descriptive statistics were shown as mean±standard deviation. The student t test was used to compare continuous variables between two groups. The difference between the groups regarding the qualitative variables was investigated with a Chi- square test. A p value less than 0.05 was considered statistically significant. Similarly, for bivariate correlation coefficients, parametric data was analyzed using Pearson's rho.

## RESULTS

Fifty-four pregnant women (age, 31.12 ± 5.72 years) who had GDM and 33 healthy pregnant women (age, 29.51 ± 4.92 years) were involved in the study. The GDM patients' and controls' demographic and biochemical characteristics are presented in Table 1. There was no significant difference in age, sex, lipid parameters, body mass index (BMI), or systolic and diastolic blood pressure. FPG, oral glucose tolerance test first hour glucose (OGTTglucose_1)_, oral glucose tolerance test second hour glucose (OGTTglucose_2_), insulin, HOMA-IR, FGF-23 levels, and mean CiMt were significantly higher in the GDM group.

**Table 1:** Characteristics of patient and control groups

Parameters	Patient group n = 54	Control group n = 33	p
Age (years)	31.12 ± 5.72	29.51 ± 4.92	0.105
BMI (kg/m^2^)	32.05 ± 5.84	30.12 ± 4.42	0.183
BSA (m^2^)	1.81 ± 0.17	1.78 ± 0.15	0.437
Systolic BP (mmHg)	110.48 ± 10.63	106.36 ± 13.09	0.113
Diastolic BP (mmHg)	69.57 ± 9.06	66.45 ± 8.76	0.055
FPG (mg/dL)	89.70 ± 12.28	75.27 ± 6.25	< 0.001
OGTTglucose_1_ (mg/dL)	189.53 ± 30.97	126.48 ± 26.22	< 0.001
OGTTglucose_2_ (mg/dL)	151.68 ± 25.78	103.33 ± 22.36	< 0.001
LDL (mg/dL)	108.98 ± 33.99	117.05 ± 33.11	0.281
HDL (mg/dL)	65.86 ± 21.60	66.21 ± 10.46	0.930
TG (mg/dL)	200.73 ± 61.05	183.21 ± 50.27	0.169
ALT (U/L)	18.64 ± 7.27	18.15 ± 6.13	0.744
Insulin (uIU/L)	12.84 ± 9.10	9.07 ± 4.99	0.032
HOMA-IR	2.71 ± 1.98	1.75 ± 1.04	0.021
Calcium (mg/dL)	8.82 ± 0.30	8.75 ± 0.27	0.284
Phosphate (mg/dL)	3.58 ± 0.41	3.75 ± 0.36	0.069
25 OH D (ng/mL)	34.31 ± 4.25	33.93 ± 3.42	0.669
PTH (pg/mL)	39.06 ± 3.38	38.67 ± 3.39	0.600
CRP	3.13 ± 0.25	3.17 ± 0.23	0.446
FGF 23 (pg/mL)	162.24 ± 22.41	68.36 ± 10.96	< 0.001
CIMT (cm)	0.064 ± 0.009	0.057 ± 0.007	< 0.001

BMI: body mass index; BSA: body surface area; BP: blood pressure; FPG: fasting plasma glucose; OGTTglucose_1_: oral glucose tolerance test first hour glucose; OGTTglucose_2_: oral glucose tolerance test second hour glucose; LDL: low density lipoprotein; HDL: high density lipoprotein; TG: triglycerides; ALT: alanine aminotransferase; HOMA-IR: homeostatic model assessment index-insulin resistance; 25 OH D: 25 hydroxy vitamin D; PTH: parathyroid hormone; FGF-23: fibroblast growth factor; CIMT: carotid intima-media thickness.

Changes in the patient group's and control group's conventional and Doppler echocardiographic measurements are presented in Table 2. LV mass and LV mass index were significantly higher in the patient group. No significant difference existed between the groups' left ventricular end-diastolic dimensions (LV EDDs), left ventricular end-systolic dimensions (LV ESDs), interventricular septum thickness (IVS), posterior wall thickness (PW), left atrial dimension, left ventricular ejection fraction (LV EF), mitral E and A velocity, E/A, DT, or LA volume index.

**Table 2:** Conventional and Doppler echocardigraphic measurements of the gestational diabetes and control groups

Parameters	Patient group n = 54	Control group n = 33	p
LV EDD (mm)	46.00 ± 3.12	44.88 ± 5.25	0.274
LV ESD (mm)	28.12 ± 6.51	28.21 ± 3.01	0.946
IVS (mm)	9.92 ± 3.83	8.60 ± 1.63	0.063
PW (mm)	13.16 ± 3.60	8.17 ± 1.47	0.384
LV mass (g)	164.75 ± 42.04	139.30 ± 29.37	0.003
LV mass index (g/m^2^)	90.47 ± 20.16	77.85 ± 15.87	0.003
Left atrial dimension (mm)	50.16 ± 11.79	48.81 ± 11.02	0.597
LV EF (%)	68.72 ± 4.74	68.63 ± 4.16	0.932
Mitral E velocity (cm/s)	83.11 ± 13.26	87.93 ± 14.59	0.117
Mitral A velocity (cm/s)	68.38 ± 11.13	71.36 ± 13.76	0.273
E/A	1.23 ± 0.24	1.25 ± 0.23	0.704
DT (ms)	170.48 ± 34.59	167.90 ± 35.23	0.739
LA volume index (mL/m^2^)	27.81 ± 6.29	27.37 ± 5.67	0.741

LV: left ventricular; LV EDD: LV end-diastolic dimension; LV ESD: LV end-systolic dimension; IVS: interventricular septum thickness; PW: posterior wall thickness; EF: ejection fraction; E: early diastolic velocity; A: late diastolic velocity; DT: mitral E-wave deceleration time; LA: left atrium.

Tissue Doppler echocardiographic parameters of the gestational diabetes and control groups are presented in Table 3. MPI was significantly higher in the patient group. HOMA-IR did not show a positive correlation with FGF-23, PW, or IVS (r = 0.309, p = 0.008; r = 0.336, p = 0.004; and r = 0.356, p = 0.002, respectively), but it showed a negative correlation with HOMA-IR and LV EDD (r = −0.242, p = 0.039). Insulin had a positive correlation with FGF-23, PW, and IVS (r = 0.271, p = 0.011; r = 0.264, p = 0.014; r = 0.282, and p = 0.008, respectively). Additionally, FPG had a positive correlation with BMI, CIMT, FGF- 23, LV mass index, PW, IVS, RVL-MPI, IVS-MPI, and LVL-MPI (r = 0.274, p = 0.010; r = 0.209, p = 0.052; r = 0.519, p < 0.001; r = 0.291, p = 0.006; r = 0.312, p = 0.003; r = 0.406, p < 0.001; r = 0.240, p = 0.025; and r = 0.285, p = 0.007, respectively). FGF-23 had a positive correlation with CIMT, LV mass index, RVL-MPI, IVS-MPI, and LVL-MPI (r = 0.400, p < 0.001; r = 0.282, p = 0.008; r = 0.544, p < 0.001; r = 0.310, p = 0.004; and r = 0.268, p = 0.012, respectively) (Table 4).

**Table 3:** Tissue Doppler echocardiographic parameters of the gestational diabetes and control groups

Parameters		Patient group n = 54	Control group n = 33	p
Left ventricular lateral wall				
	Sm (cm/s)	10.75 ± 2.61	10.42 ±1.71	0.514
	Em/Am	1.49 ± 0.42	1.34 ± 0.68	0.211
	MPI	0.50 ± 0.94	0.44 ± 0.08	0.004
Interventricular septum				
	Sm (cm/s)	8.22 ± 1.65	9.15 ± 2.07	0.024
	Em/Am	1.28 ± 0.41	1.13 ± 0.55	0.154
	MPI	0.52 ± 0.07	0.42 ± 0.09	0.002
Right ventricular lateral wall				
	Sm (cm/s)	16.48 ± 3.73	18.87 ± 5.12	0.014
	Em/Am	0.97 ± 0.35	0.84 ± 0.43	0.128
	MPI	0.55 ± 0.09	0.41 ± 0.07	< 0.001

Sm: systolic myocardial velocity; Em: myocardial early diastolic velocity; Am: myocardial late diastolic velocity; MPI: myocardial performance index.

**Table 4:** Correlation between metabolic parameters, FGF-23, convensional and tissue doppler echocardiographic parameters of the all participants

Parameters		HOMA-IR	Insulin	FPG	OGTTglucose_1_	OGTTglucose_2_	FGF-23
BMI (kg/m^2^)							
	r	0.048	0.025	0.274	0.075	0.234	0.181
	p	0.686	0.817	0.010	0.489	0.029	0.094
CIMT (cm)							
	r	0.136	0.126	0.209	0.339	0.156	0.400
	p	0.251	0.244	0.052	0.001	0.150	< 0.001
FGF-23							
	r	0.309	0.271	0.519	0.631	0.679	–
	p	0.008	0.011	< 0.001	< 0.001	< 0.001	–
LVMI (g/m^2^)							
	r	0.097	0.076	0.291	0.180	0.262	0.282
	p	0.414	0.482	0.006	0.095	0.014	0.008
E/A							
	r	0.045	0.052	-0.086	-0.090	-0.070	-0.030
	p	0.707	0.632	0.430	0.407	0.517	0.783
LV mass (g)							
	r	0.159	0.109	0.312	0.178	0.259	0.298
	p	0.178	0.316	0.003	0.099	0.016	0.005
RVL-MPI							
	r	0.196	0.143	0.406	0.455	0.464	0.544
	p	0.096	0.187	< 0.001	< 0.001	< 0.001	< 0.001
IVS-MPI							
	r	0.087	0.153	0.240	0.230	0.172	0.310
	p	0.462	0.158	0.025	0.032	0.110	0.004
LVL-MPI							
	r	0.032	0.031	0.285	0.244	0.248	0.268
	p	0.788	0.774	0.007	0.023	0.021	0.012

BMI: body mass index; CIMT: carotid intima-media thickness; FGF-23: fibroblast growth factor 23; LVMI: left ventricular mass index; E: mitral early diastolic velocity; A: mitral late diastolic velocity; RVL: right ventricular lateral wall; LVL: left ventricular lateral wall; MPI: myocardial performance index; HOMA-IR: homeostatic model assessment index-insulin resistance; FPG: fasting plasma glucose; OGTTglucose_1_: oral glucose tolerance test first hour glucose; OGTT glucose_2_: oral glucose tolerance test second hour glucose

## DISCUSSION

Several previous studies demonstrated that FGF-23 is independently associated with several cardiovascular risk factors, such as endothelial dysfunction, increased arterial stiffness, LVH, left ventricular dilatation impaired vasoreactivity, cardiovascular mortality, and atherosclerosis in hemodialysis patients and healthy subjects (14,15). CIMT assessment and common carotid artery plaque identification by ultrasound are well-recognized tools for identification and monitoring of atherosclerosis. Elevated serum FGF-23 levels have been found to be associated with vascular diseases such as CIMT, arterial stiffness, and coronary atherosclerosis in patients with advanced CKD (16,17). Although Balci and cols. (19) reported a positive correlation between CIMT and FGF-23 in hemodialysis patients, Gungor and cols. (20) did not find any correlation. Several studies have indicated that a pregnancy complicated by GDM has a significant impact on endothelial function during pregnancy (21,22). However, the impact of FGF-23 on the early stages of atherosclerosis in patients with gestational diabetes is not defined. In this study, mean CIMT was significantly higher in patients with GDM, and a positive correlation between FGF-23 and CIMT was found. Association of FGF-23 with CIMT may be another possible mechanism underlying the development and progression of atherosclerosis in patients with GDM.

The myocardial performance index reflects LV systolic and diastolic function. MPI is also abnormal in individuals without overt cardiac disease who have risk factors such as diabetes mellitus and treated and untreated hypertension (17,23,24). In a recent study, MPI was measured by the conventional method and with tissue Doppler echocardiography. It was significantly higher in the diabetic group than in the control group (25). In patients with CKD, FGF-23 supraphysiologic levels were strongly associated with LVMI, left ventricular hypertrophy (LVH), cardiovascular events, and mortality (14,26). High prevalence of preclinical diastolic dysfunction is a well-established fact among diabetic patients. The evidence indicates that myocardial damage in diabetic subjects affects diastolic function before the systolic function (27). Using tissue Doppler and strain/strain rate echocardiography, a recent study showed that LV longitudinal systolic and diastolic functions were impaired in diabetic patients (28). However, limited studies have been conducted on GDM's effect on diastolic functions (29). Caliskan and cols. (29) found that although LV systolic function and LVMI were similar between the two groups (GDM and control groups), the diastolic function parameters were impaired in the GDM group. In our study, although LVMI was significantly higher in the GDM group than in the control group, no significant difference existed in diastolic function parameters (mitral E/A ratio) between groups. Sm determined by TDI is another measure of LV systolic functions independent from the LV's shape (31). In our study, Sm had fewer patients with GDM, indicating the presence of possible subclinical systolic dysfunction. MPI was significantly higher in patients with GDM than in the control group. Additionally, FGF-23 showed a significant positive correlation with MPI. Therefore, we think MPI, which reflects diastolic and systolic functions, is a more important marker than mitral inflow velocity (E/A) in the evaluation of cardiac functions.

The data concerning the relationship between FGF-23 levels and IR are contradictory. Wahl and cols. (13) have observed that FGF-23 levels are greater in coronary artery disease patients who have diabetes. Garland and cols. (30) reported that increasing HOMA-IR was positively associated with FGF-23 in stage 3-5 chronic kidney disease patients. Holecki and cols. (31) showed increased levels of both circulating FGF-23 forms in elderly subjects are not associated with obesity or IR. Hanks and cols. (32) found that the surrogate marker of IR, HOMA-IR were positively associated with FGF-23. The relationship between IR and FGF-23 has not been investigated in patients with GDM. This study showed a positive correlation between HOMA-IR and FGF-23. Association of FGF-23 with IR may be another possible mechanism underlying the development and progression of atherosclerosis in DM patients.

In conclusion, the present prospective study is the first in the literature that evaluated the relationship between FGF-23 and MPI in patients with gestational diabetes in the absence of CVD. Plasma FGF-23 levels were shown to be associated with increased LVMI, MPI, and CIMT in patients with GDM. The significant and positive association of FGF-23 plasma level with LVMI and MPI raises the possibility of a specific pathophysiologic effect of FGF-23 on left ventricular mass and function distinct from its effects on serum calcium, phosphorus, and intact parathormone. Additionally, this study showed that FGF-23 may play a role in the development of GDM with IR. Therefore, we believe that FGF-23 plays a role in the pathogenesis and development of preclinical atherosclerosis and cardiac dysfunction in patients with GDM.
